# Effects of heart rate variability biofeedback during exposure to fear-provoking stimuli within spider-fearful individuals: study protocol for a randomized controlled trial

**DOI:** 10.1186/s13063-018-2554-2

**Published:** 2018-03-16

**Authors:** Sarah K. Schäfer, Frank R. Ihmig, Karen A. Lara H., Frank Neurohr, Stephan Kiefer, Marlene Staginnus, Johanna Lass-Hennemann, Tanja Michael

**Affiliations:** 1 Division of Clinical Psychology and Psychotherapy, Department of Psychology, Saarland University, Building A 1.3, 66123 Saarbrücken, Germany; 2 Department of Biomedical Microsystems, Fraunhofer-Institut fuer Biomedizinische Technik (IBMT), Sulzbach, Saar Germany

**Keywords:** HRV biofeedback, Heart rate variability, Biofeedback, Fear of spiders, Spider phobia, Exposure therapy, Exposition therapy

## Abstract

**Background:**

Specific phobias are among the most common anxiety disorders. Exposure therapy is the treatment of choice for specific phobias. However, not all patients respond equally well to it. Hence, current research focuses on therapeutic add-ons to increase and consolidate the effects of exposure therapy. One potential therapeutic add-on is biofeedback to increase heart rate variability (HRV). A recent meta-analysis shows beneficial effects of HRV biofeedback interventions on stress and anxiety symptoms. Therefore, the purpose of the current trial is to evaluate the effects of HRV biofeedback, which is practiced before and utilized during exposure, in spider-fearful individuals. Further, this trial is the first to differentiate between the effects of a HRV biofeedback intervention and those of a low-load working memory (WM) task.

**Methods:**

Eighty spider-fearful individuals participate in the study. All participants receive a training session in which they practice two tasks (HRV biofeedback and a motor pseudo-biofeedback task or two motor pseudo-biofeedback tasks). Afterwards, they train both tasks at home for 6 days. One week later, during the exposure session, they watch 16 1-min spider video clips. Participants are divided into four groups: group 1 practices the HRV biofeedback and one motor pseudo-task before exposure and utilizes HRV biofeedback during exposure. Group 2 receives the same training, but continues the pseudo-biofeedback task during exposure. Group 3 practices two pseudo-biofeedback tasks and continues one of them during exposure. Group 4 trains in two pseudo-biofeedback tasks and has no additional task during exposure. The primary outcome is fear of spiders (measured by the Fear of Spiders Questionnaire and the Behavioral Approach Test). Secondary outcomes are physiological measures based on electrodermal activity, electrocardiogram and respiration.

**Discussion:**

This RCT is the first one to investigate the effects of using a pre-trained HRV biofeedback during exposure in spider-fearful individuals. The study critically contrasts the effects of the biofeedback intervention with those of pseudo-tasks, which also require WM capacity, but which do not have a physiological base. If HRV biofeedback is effective in reducing fear of spiders, it would represent an easy-to-use tool to improve exposure-therapy outcomes.

**Trial registration:**

Deutsches Register Klinischer Studien, DRKS00012278. Registered on 23 May 2017, amendment on 5 October 2017.

**Electronic supplementary material:**

The online version of this article (10.1186/s13063-018-2554-2) contains supplementary material, which is available to authorized users.

## Background

Specific phobias are characterized by an excessive and irrational fear of a specific object or situation, which is either avoided or endured with great distress. About 12.5% of the population meet the criteria for a specific phobia at least once in their lifetime [1]. Current theories on the pathogenesis of anxiety disorders and phobias have been strongly influenced by models of conditioning and associative learning [2, 3]. In phobic individuals, exposure to the fear-provoking stimulus triggers the retrieval of stimulus-related fear memories, which, in turn, lead to the fear response [4–6]. One central mechanism of exposure therapy, the treatment of choice for specific phobias, is the extinction of these fear responses. This is accomplished by exposing the patient repeatedly and systematically to these stimuli under controlled conditions [7–9]. Even though exposure therapy is the first-line treatment for anxiety disorders, not all patients benefit equally from it. Some achieve only partial remission or show a return of fear after an initial therapeutic success [8–10]. Thus, one of the main aims of current research on the treatment of anxiety disorders is to develop ways of increasing and consolidating the effects of exposure therapy [11, 12]. A biofeedback intervention to enhance heart rate variability (HRV) is currently discussed as a particular promising therapeutic add-on [13, 14].

Several studies show that HRV as a biomarker of autonomous nervous system (ANS) functioning is linked to physical and mental health: serious psychological disorders, such as depression [15] and anxiety disorders [16], are associated with a substantially reduced HRV. This reduction is also linked to decreased motivation to engage in social situations, self-regulation problems, and less flexible reactions to psychological stressors [17]. Moreover, in the long term, immune dysfunctions, inflammatory reactions, cardiovascular diseases and higher mortality are associated with a decreased HRV [18].

However, thus far, it is not known which mechanisms link HRV to health and illness. The heart is innervated equally by both the sympathetic and parasympathetic ANS paths. HRV can be considered as a dynamic index of their interdependency. However, parasympathetic (vagal) influences have the greater and faster impact on HRV [17]. Further, a meta-analysis based on neuroimaging studies suggests that HRV reflects not only the relation of sympathetic and parasympathetic ANS paths, but also an individual’s ability to adapt to environmental demands [18]. Hence, a greater HRV reflects an individual’s capability to deal with demands in a flexible way, while a smaller HRV corresponds to a limited repertory of behavioral responses.

Irrespective of its precise mode of action (see [19] for different theories), several studies report beneficial effects of HRV biofeedback [13]. The intervention is generally conceptualized as a biofeedback of respiratory sinus arrhythmia (RSA) that reflects the variation in heart rate (HR) which accompanies breathing. Participants are instructed to synchronize their breathing frequency with their oscillations in HR. This intervention is commonly and, therefore also hereafter, called HRV biofeedback, although technically it is not a genuine feedback of HRV, but a feedback of HR [20]. During standard HRV biofeedback, participants are asked to inhale while their HR increases and to exhale while it decreases. They should breathe slowly in order to reach their individual resonance frequency at which their RSA is at maximum. On average this frequency is reached at about six breaths per min [21]. Particularly well-elaborated versions of HRV biofeedback contain several visually complex graphical illustrations of HR oscillations, respiratory rate and RSA (e.g., [20]).

Regarding the effectiveness of HRV biofeedback, a recent meta-analysis based on clinical and non-clinical samples reports beneficial effects of HRV biofeedback as a stand-alone intervention on symptoms of stress and anxiety [22]. HRV biofeedback significantly reduces stress and anxiety symptoms (*g* = .81) and is superior to various control conditions such as waiting list of sham biofeedback (*g* = .83). Furthermore, several studies have evaluated the effects of HRV biofeedback as add-on intervention in clinical samples: in posttraumatic stress disorder, a case study and a controlled trial have shown beneficial effects of HRV biofeedback during exposure therapy [23, 24]. Positive results in terms of reduced depression scores and increased HRV have also been shown for patients with depression [25, 26]. Further, a recent pilot study demonstrated a trend towards an increased fear reduction in aviophobia patients who received diaphragmatic breathing training prior to exposure in virtual reality [27]. The training used a frequency of six breath cycles per min, which is equal to the average resonance frequency normally used in HRV biofeedback interventions to maximize individual HRV. Hence, it is plausible to assume that the reported beneficial effects are linked to HRV increase caused by slow diaphragmatic breathing.

Taken together, current evidence suggests that HRV biofeedback may be a useful stand-alone intervention and a promising add-on tool to improve psychotherapy outcomes. However, thus far, there is a lack of randomized controlled trials (RCT) supporting the effectiveness of the intervention. This is critical for two reasons: firstly, testing the effects of a therapeutic add-on in a controlled trial is necessary in terms of advancing evidence-based treatments. Secondly, the effects of HRV biofeedback need to be disentangled from possible effects of working memory (WM) tasks, as different studies demonstrate a positive impact of low-load WM tasks on consolidation and reconsolidation memory processes relevant for memorizing therapeutic input [28, 29]. For example, theories on the mechanisms underlying Eye Movement Desensitization and Reprocessing (EMDR) emphasize the role of eye movements as a low-load WM-demanding task [30, 31]. Performing eye movements during imagery exposure reduces WM capacity, thereby decreasing the vividness and emotionality of the traumatic memory [32].

### Aim of the current trial

**Fig. 1:**
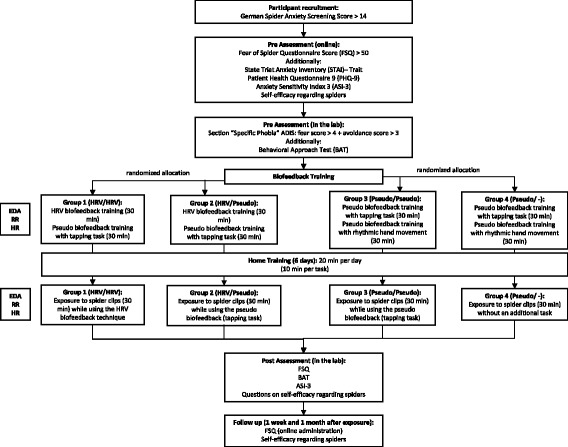
Experimental flow chart including all trial phases

While HRV biofeedback is not conceptualized as a WM task, it nonetheless creates a WM load that might influence relevant (re-)consolidation processes during exposure. Therefore, the current trial aims to examine whether HRV biofeedback is more effective than pseudo-biofeedback that evokes a similar WM load. All experimental groups receive a training session during which they learn two tasks (either HRV biofeedback and a pseudo-biofeedback task or two pseudo-biofeedback tasks). Both pseudo-biofeedback tasks require the participants to synchronize a repetitive tapping on a table (A) or a rhythmic hand movement (B) with a pseudo-physiological signal. After one week of training at home, all participants return to the laboratory and watch a series of spider video clips. Group 1 (G1, HRV/HRV) is trained in both HRV biofeedback and in pseudo-biofeedback task A (1), but uses HRV biofeedback during exposure. Group 2 (G2, HRV/pseudo) receives the same training as G1, but performs the pseudo-biofeedback task A during exposure. Group 3 (G3, pseudo/pseudo) is trained in two pseudo-biofeedback tasks (A and B) and uses the same pseudo-biofeedback task A as group 2 during exposure. Group 4 (G4, pseudo/−) is trained in both pseudo-biofeedback tasks (A and B), but performs none of them during exposure (for a detailed overview, see Fig. 1).

### Hypotheses

Based on prior studies on HRV biofeedback and findings on the impact of WM tasks on exposure therapy, it is hypothesized that:

1. Participants who are trained in HRV biofeedback, and who utilize it during exposure, have the greatest reduction in fear.
2. Participants who are trained in HRV biofeedback, but who do not utilize it during exposure, have more fear reduction than participants who are not trained in HRV biofeedback.
3. Participants who utilize a WM task (pseudo-biofeedback task A) during exposure show greater fear reduction than participants who do not have an additional task during exposure.

Further, we will analyze physiological fear measurements [electrodermal activity (EDA), electrocardiogram (ECG) and respiration (RSP)] during biofeedback training and exposure sessions and relate changes in these signals to changes in fear.

## Methods

### Participants

Participation is limited to spider-fearful participants aged 18 to 40 years. Participants are regarded as spider-fearful individuals (SFs) when they score 14 or more in the German Spider Anxiety Screening (SAS, [33]), which is the commonly used cut-off score [34, 35]. Moreover, they have to score 50 or higher on the Fear of Spiders Questionnaire (FSQ [36]; German version: [33]). Additionally, SFs must reach a minimum fear score of 4 and an avoidance score of 3 in the section “Specific Phobias” of the structured interview for mental disorders (ADIS – section “Specific Phobias” [39]; German version: [40]). Exclusion criteria are: presence of any other mental disorder than spider phobia (assessed with the Patient Health Questionnaire D (PHQ-D, [37]) and the Beck Depression Inventory (BDI-II; German version: [38]) and any reported cardiovascular disease (assessed during the screening phase).

Recruiting takes place at Saarland University, in social media and through the local press. Participants are contacted individually by the research team and provide informed consent prior to their participation. Psychology students taking part in the study receive course credit.

### Sample size

The intended samples size was calculated using G*Power 3.1 [39]. An a priori power calculation (*α* = .05, 1 – *β* = .85) based on the weighted mean between-group effect reported by Goessl et al. [22] resulted in a total sample size of 80 participants. Thus, 20 participants per experimental group are recruited to ensure sufficient statistical power.

### Materials and measures

#### Interview

##### Anxiety Disorders Interview Schedule for DSM–IV

The Anxiety Disorder Interview Schedule for the Diagnostic and Statistical Manual of Mental Disorders IV text revised (DSM-IV-TR) (ADIS; [40]) is a structured interview which relies on the diagnostic criteria of the DSM-IV-TR. In the current study, the section “Specific Phobias” of the German ADIS version DIPS (Diagnostisches Interview bei Psychischen Störungen, [41]) is used to assess the participants’ fear and avoidance behavior towards spiders.

#### Subjective ratings – screening

##### German Spider Anxiety Screening

The SAS [33] is a four-item self-report questionnaire to efficiently screen SFs in larger samples. Scores range from 0 to 24 with higher values indicating a stronger fear of spiders. In the present trial, the screening is performed by a trained member of the research team using a phone version of the SAS.

##### Fear of Spiders Questionnaire (FSQ)

The FSQ ([36]; German version: [33]) is a self-report instrument to quantify fear of spiders. It consists of 18 items which are rated on a 7-point scale ranging from “1 = not at all” to “7 = very much”. The questionnaire shows good psychometric properties [33, 36].

##### Anxiety Sensitivity Scale 3

The Anxiety Sensitivity Scale (ASI-3; [42]; German version: [43]) is administered to measure fearful cognitions about physiological anxiety symptoms. The 16-item scale is used to control for a priori differences between the four experimental groups.

##### Beck Depression Inventory II

To assess depressive symptoms within the last two weeks, the German version of the Beck Depression Inventory (BDI-II; German version: [38]) is used. It contains 21 items related to depression with scores ranging from 0 to 63. Scores of 17 and higher are considered clinically relevant. Participants scoring 17 or higher are excluded.

##### Patient Health Questionnaire D

The Patient Health Questionnaire D (PHQ-D; [44], German version: [37]) is a short and economic instrument for assessing symptoms of mental health disorders. Its newest version relies on the DSM-IV-TR criteria and has good psychometric properties [45].

#### Subjective ratings – outcome

##### Fear of Spiders Questionnaire

The FSQ ([36]; German version: [33]) is not just used as a screening tool, but also for outcome assessment. Differences in FSQ scores from pre to post treatment and to follow-up are used as the primary outcome measure.

##### Self-efficacy concerning spiders

In accordance with Shiban et al. [27], three questions on the perceived efficacy in handling a spider are asked (see Additional file 1).

##### STAI-S and STAI-T

The State and Trait Anxiety Inventory (STAI; [46]; German version: [47]) is used to assess subjective baseline anxiety (trait scale) as well as short-term changes in anxiety levels (state scale). Both STAI versions are brief self-report questionnaires consisting of 20 items related to perceived nervousness, tension and worry. All items are rated on a 4-point scale. Hence, total STAI scores range from 20 to 80, with higher scores indicating higher anxiety levels. Both scales show good psychometric properties [47].

##### Subjective stress ratings

After every fourth video clip in the exposure session, participants are asked to rate their subjective fear and arousal levels on 4-point scales from “1 = not at all” to “4 = strongly”.

#### Behavioral test

##### Behavioral Approach Test

To test participants’ fear and avoidance behavior towards real spiders, the Behavioral Approach Test (BAT; [48]) is employed. The BAT (as administered in a prior study by Lass-Hennemann and Michael [49]) consists of the following procedure: standing in front of a closed room that contains a house spider (*Tegenaria atrica*) measuring about 5 cm (legs included). The spider is placed in a sealed plastic container on a table at the end of the room. Next, if possible, the participant enters the room, approaches the container, removes the lid, inserts a hand and tries to pick up and hold the spider for at least 20 s. When the participant has reached for, or touched, the spider, or when they decide to stop approaching, the remaining distance is noted. In detail, 13 steps are coded: 0 = test room is not entered; 1 = stops 5 m from the container, 2 = stops 4 m from the container; 3 = stops 3 m from the container; 4 = stops 2 m from the container, 5 = stops 1 m from the container, 6 = stops close to the table with the container, 7 = touches the container, 8 = removes the lid, 9 = puts a hand in the container, 10 = touches the spider with one finger, 11 = holds the spider less than 20 s, 12 = holds the spider for at least 20 s. Differences from pre to post treatment are used as a primary outcome measure.

#### Physiological stress measurement

**Fig. 2:**
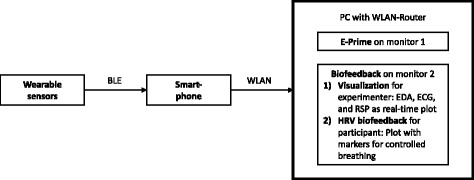
Schematic diagram of the Biofeedback System

Physiological data is recorded using the Biofeedback System (BFS) developed by Fraunhofer IBMT. This system supports state-of-the-art wearable sensors and wireless communication to enhance usability and the participant’s comfort. The schematic diagram (see Fig. 2) shows the set-up of the BFS.

EDA, ECG and RSP signals are recorded using the BITalino biosignal measurement device (PLUX – Wireless Biosignals S.A., Lisbon, Portugal) with the sampling frequency set to 100 Hz per channel, which is sufficient for ECG rhythm monitoring [50]. Three electrodes are placed according to standard lead II configuration. For EDA measurement two electrodes are attached to the proximal part of the palm of the participant’s non-dominant hand. The electrodes used are standard pre-gelled and self-adhesive disposable Ag/AgCl electrodes (Kendall H135SG, Medtronic, Minneapolis, MN, USA). The BITalino RSP sensor is an adjustable, elastic-fastening chest strap with an integrated piezoelectric sensor. HRV biofeedback is obtained by employing the HR monitor armband Rhythm+ (Scosche Industries, Inc., Oxnard, CA, USA) with a sampling frequency of 1 Hz. This wristband is placed below the elbow of the participant’s non-dominant arm. All sensor data is wirelessly routed to the personal computer (PC) through a smartphone (Nexus 5, Google Inc., Mountain View, CA, USA) using BLE (Bluetooth Low Energy) and WLAN (Wireless Local Area Network) interfaces.

The PC software used for data acquisition and visualization has been developed in C# (Visual Studio Enterprise 2015, Microsoft Corporation, Redmond, WA, USA) as a Windows Form application. This application plots real-time physiological signals and saves them into text files. Additionally, the PC software plots the visualization of the HRV biofeedback and pseudo-biofeedback. Since all instructions are presented to the participants by using E-Prime software (E-Prime 2.0, Psychology Software Tools Inc., Sharpsburg, MD, USA), the PC application incorporates an interface for receiving trigger signals related to defined events during the training and exposure sessions.

#### Tasks

##### Biofeedback trainings

The biofeedback training session takes place in the laboratory. Participants are seated in front of a computer monitor, which displays all instructions. The biofeedback information is displayed on a second monitor (see a photograph of the experimental set-up as Additional file 2).

##### HRV biofeedback

The training session consists of a verbal instruction to synchronize one’s breathing and the displayed changes in HR. All instructions are adapted from a well-established HRV biofeedback training provided by Lehrer et al. [20]. Participants are asked to inhale when HR is increasing, and to exhale while it is decreasing. The visualization of the biofeedback signal is similar to the commercial StressEraser® device [51]. Further, the training includes two schematic diagrams of the simultaneously displayed HRV biofeedback and the correct breathing rhythm (see as Additional file 3). Neither the schematic nor the real HR diagrams contain absolute HR values in order to prevent the participants from focusing on their HR levels.

##### Resonance frequency

The BFS obtains and displays the current breathing frequency (breaths per min) based on a “peaks and valleys detection” algorithm. The breathing frequency is calculated as the number of detected peaks in a period of 1 min.

##### Pseudo-biofeedback

The pseudo-biofeedback training also uses verbal instructions and a schematic diagram of the intended training results (see as Additional file 3). In contrast to the HRV biofeedback, participants are asked to synchronize a specific movement (tapping or a smooth, rhythmic hand movement) to the displayed signal. In order to illustrate the intended movements, short demonstration video clips are presented. The pseudo-signal is introduced as “oxygen variability” and changes over time to match the changes in HR due to the breathing instruction.

##### Pseudo-biofeedback signal

The signal that emulates the oxygen variability is implemented using the following equation (Eq. 1):

$$ f(t)\kern0.5em =\kern0.5em \left\{\begin{array}{c}A\cdot \sin \left(\frac{\pi }{T_{\mathrm{peak}}}t\right)+0.3\kern0.2em N,\kern1.5em if\kern0.5em 0\kern0.5em \le t\le \frac{T_{\mathrm{peak}}}{2}\kern10em \\ {}A\cdot \sin \left(\frac{\pi }{T_{\mathrm{peak}}}t\right)-0.4\kern0.2em N,\kern1.5em if\frac{T_{\mathrm{peak}}}{2}\kern0.5em <t\le {T}_{\mathrm{peak}}\kern8em \\ {}0,\kern15em if\kern0.2em {T}_{\mathrm{peak}}\kern0.5em <\mathrm{t}<{T}_{\mathrm{peak}}+{T}_{\mathrm{non}-\mathrm{peak}}\end{array}\right. $$

where *T*_peak_ is a random number between 4 and 7, *N* is a random number between 0 and 1, *A* is the amplitude that starts with 6 and, after 5 min, changes to 8. *T*_non-peak_ is a random number between 3 and 5. Every time *t* reaches *T*_peak_ + *T*_non-peak_, *t* is set to 0 and new values for the periods are calculated. Using this equation a pseudo-signal is generated which looks similar to the HRV biofeedback signal.

##### Practice at home

At the end of the training sessions, all participants receive an MP3 file to continue their training at home. This file contains an audio instruction for a 20-min session, during which each type of training (HRV and/or pseudo-biofeedback) is practiced for 10 min. All instructions ask the participant to synchronize their breathing or a movement to a repeatedly presented, pleasant audio sound. In case of the HRV biofeedback, its frequency corresponds to the individual resonance frequency, which is determined by the end of the initial training session. For both pseudo-biofeedback tasks the frequency is set to a fixed value. To ensure that participants have completed the training at least once, all instruction versions contain a code word, which participants are asked to report after the exposure session.

##### Exposure session

**Fig. 3:**
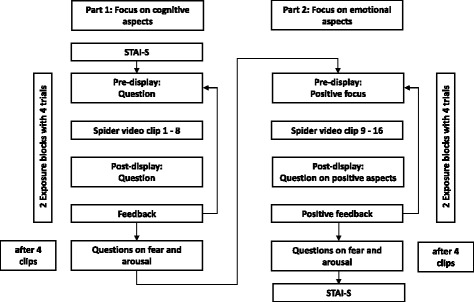
Schematic illustration of the exposure session

Firstly, all participants are introduced to the exposure procedure and rationale, which is mainly based on the principles of the one-session exposure treatment developed by Öst [52]. Its key aspects are controlled exposure to the fear-provoking stimulus and changes of fearful cognitions. Every exposure trial starts with a question on the content of the video clip (e.g., how many spiders are shown in the next clip?) in order to set a cognitive focus for the following clip. The 16 1-min clips, all taken from TV documentaries, show detailed shots of spiders. After every clip, the question asked prior to the clip is presented again and participants are asked to choose the right answer by choosing between four possible multiple choice answers. In case of a correct answer, the participant is praised. In case of an incorrect response, an instruction reminding the participants to concentrate on the videos in order to reduce their fear is presented. After eight of the 16 clips, the questions focus on positive emotional features instead of cognitive aspects to change the participant’s attitude towards spiders. Participants are constantly complimented on identifying positive facets. See Fig. 3 for a detailed illustration of the exposure procedure.

### Procedure and design

**Fig. 4:**
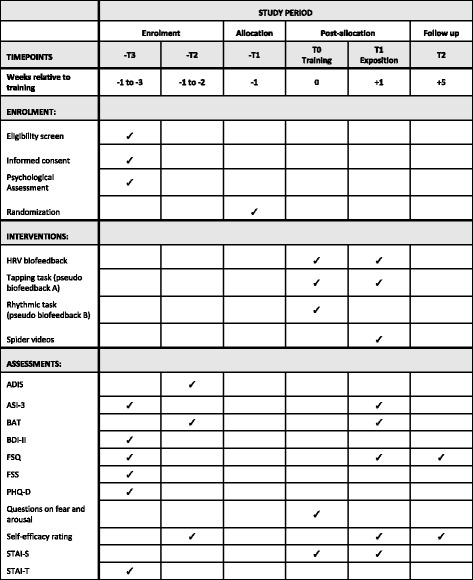
Standard Protocol Items: Recommendations for Interventional Trials (SPIRIT) Figure of the current trial

The trial takes place at the Department of Clinical Psychology and Psychotherapy at Saarland University. It includes seven phases: initial screening and diagnostic phase, randomization, (pseudo)-biofeedback training, practice at home, exposure to spider video clips, post-assessment and follow-up assessments. For a detailed temporal overview see the Standard Protocol Items: Recommendations for Interventional Trials (SPIRIT) Figure (Fig. 4). The SPIRIT Checklist is provided as Additional file 4.

#### Screening and diagnostic phase

Participants who volunteered to take part in the trial are contacted by phone for a short screening using the SAS [33]. Participants scoring 14 or higher are invited to complete an online questionnaire administered via SoSci Survey [53], which consists of the STAI-T, the FSQ, the PHQ-D, the ASI-3, the BDI-II and three questions on the perceived self-efficacy in handling spiders. If participants reach a FSQ score of 50 or higher, they are asked to attend an assessment at the Department of Clinical Psychology and Psychotherapy. This first assessment takes about 45 min and contains the ADIS section “Specific Phobias” and the BAT. Both are administered by a trained member of the research team. Participants with an avoidance score of at least three and a fear score of at least four are invited to take part in the biofeedback training.

#### Randomization

The randomization process using a generated list is conducted by a member of the research team who is not involved in the data collection process. Participants are allocated to one of the four aforementioned experimental groups (*n* = 20 per group): (1) group 1 (G1, HRV/HRV); (2) group 2 (G2, HRV/pseudo); (3) group 3 (G3, pseudo/pseudo); and (4) group 4 (G4, pseudo/−).

#### Biofeedback training

The biofeedback training session starts with a resting phase of 5 min to assess baseline HRV. Afterwards, participants complete the STAI-S. Then, depending on the experimental group, the training session starts. All participants receive two types of training, each lasting 30 min. The order of both training parts is randomized within every group. After each type of training, participants complete the STAI-S again to evaluate their current anxiety level.

#### Practice at home

At the end of the training session, all participants receive an audio file for home training. The version of the provided file depends on the participants’ individual resonance frequency (G1 and G2) or takes the form of a standard audio file for both pseudo-biofeedback tasks (G3 and G4). Participants are asked to continue the training for 6 days (20 min a day).

#### Exposure session

The exposure session is conducted one week after the initial training session in the same laboratory using the same technical devices. All exposure sessions take place in the afternoon (between 2 p.m. and 6 p.m.) to control for diurnal cortisol levels, which can influence exposure effects [49]. At first, all participants complete the STAI-S. Then participants go on to conduct exposure. G1 receives HRV biofeedback during exposure, whereas G2 and G3 are presented with pseudo-biofeedback (task A). G4 has no additional task during exposure.

#### Post assessment

Subsequent to the exposure session, the post assessment takes place: participants complete the STAI-S, the FSQ and the ASI-3. Again, they answer three questions on their perceived self-efficacy in dealing with a spider and the post-exposure BAT is performed.

#### Follow-up assessments

One week and one month after the exposure session, participants are contacted by the research team and asked to complete an online version of the FSQ and to answer the questions on self-efficacy in handling a spider.

### Data collection and statistical analyses

Questionnaire data is registered using SoSci Survey [53] and exported as a .csv file. The BAT results are noted in an Excel sheet by a member of the research team. Further, behavioral data concerning the training and exposure sessions is collected using the E-Prime software and physiological data is stored as a text file using the BFS. All statistical analyses will be conducted by a member of the research team, who was not involved in the data collection process, using IBM SPSS version 24 [54]. The alpha level will be set to *p* < 0.05. The main hypotheses will be tested using a mixed analysis of variance (ANOVA) with group as the between-subjects factor and time (pre assessment, post assessment, follow-up one week, follow up four weeks) as the within-subjects factor. All analyses will be reported with partial *η*^*2*^ as the effect size.

## Discussion

The results of the current trial should provide evidence on the effectiveness of HRV biofeedback as a tool to enhance exposure-therapy outcome. In contrast to existing studies, the current study carefully compares the effects of a HRV biofeedback intervention with those of a low-load WM-demanding task during exposure. Moreover, the study relates exposure-evoked changes to alterations in physiological signals during exposure and to the signals recorded during the prior training session. If HRV biofeedback is found to be beneficial, monitored physiological changes might provide important information on the mechanisms underlying these effects. Finally, the association between physiological fear correlates and subjectively perceived fear and arousal is examined at four points during exposure. This is highly relevant as prior studies on this relationship have shown inconsistent findings [55, 56].

However, it has to be noted that the current trial uses a subclinical sample. In case of promising findings, further studies need to examine if HRV biofeedback interventions also improve the effectiveness of state-of-the-art psychotherapy in clinical samples. Overall, the current RCT is a first step of a detailed evaluation process aiming to improve exposure therapy by using HRV biofeedback techniques.

## Trial status

At the time of initial manuscript submission, recruitment was already in progress (since June 2017) but not yet complete (prospective completion in May 2018). The manuscript reports protocol version 2 (5/10/2017).

## Additional files

Additional file 1: Questions on self-efficacy in handling a spider. (PDF 25 kb)

Additional file 2: Photograph of the experimental set-up. (PDF 13386 kb)

Additional file 3: Schematic diagram of heart rate variability (HRV) biofeedback and pseudo-biofeedback tasks. (PDF 387 kb)

Additional file 4: SPIRIT Checklist. (DOC 122 kb)

## Abbreviations

ADIS: Anxiety Disorder Interview Schedule for DSM-IV
ANS: Autonomous nervous system
ASI-3: Anxiety Sensitivity Index 3
BAT: Behavioral Approach Test
BDI-II: Beck Depression Inventory II
BLE: Bluetooth Low Energy
DSM-IV-TR: Diagnostic and Statistical Manual of Mental Disorders IV text revised
ECG: Electrocardiogram
FSQ: Fear of Spiders Questionnaire
HR: Heart rate
HRV: Heart rate variability
PC: Personal Computer
PHQ-D: Patient Health Questionnaire 9 - German version
RCT: Randomized controlled trial
RSP: Respiration
SAS: German Spider Anxiety Screening
SF: Spider-fearful individual
STAI-S/-T: State Trait Anxiety Inventory State or Trait
WLAN: Wireless Local Area Network
WM: Working memory
