# Supplementary material for: Effects of heart rate variability biofeedback during exposure to fear-provoking stimuli within spider-fearful individuals: study protocol for a randomized controlled trial
Source: Trials. 2018 Mar 16;19:184. doi: 10.1186/s13063-018-2554-2 (PMC5857097; doi:10.1186/s13063-018-2554-2)
Supplement: Supplementary file 1 — Questions on self-efficacy in handling a spider. (PDF 25 kb) [file 13063_2018_2554_MOESM1_ESM.pdf]

ADDITIONAL FILE 1: Effects of Heart Rate Variability Biofeedback during Exposure to Fear-Provoking Stimuli within Spider Fearful Individuals: Study Protocol for a Randomized-Controlled Trial

AUTHORS: Sarah K. Schäfer, Frank R. Ihmig, Karen A. Lara H., Frank Neurohr, Stephan Kiefer, Marlene Staginnus, Johanna Lass-Hennemann & Tanja Michael

Questions on self-efficacy in handling a spider (all rate one a scale from 0 "totally unconfident" to 10 "totally confident"):

1. How confident do you feel at the moment in/about dealing with a spider?

2. How confident do you feel at the moment about being in a room, in which you know is a spider?

3. How confident do you feel at the moment of/about catching a spider with a glass in your apartment?
