# Supplementary figures and images for: Effects of heart rate variability biofeedback during exposure to fear-provoking stimuli within spider-fearful individuals: study protocol for a randomized controlled trial
Source: Trials. 2018 Mar 16;19:184. doi: 10.1186/s13063-018-2554-2 (PMC5857097; doi:10.1186/s13063-018-2554-2)

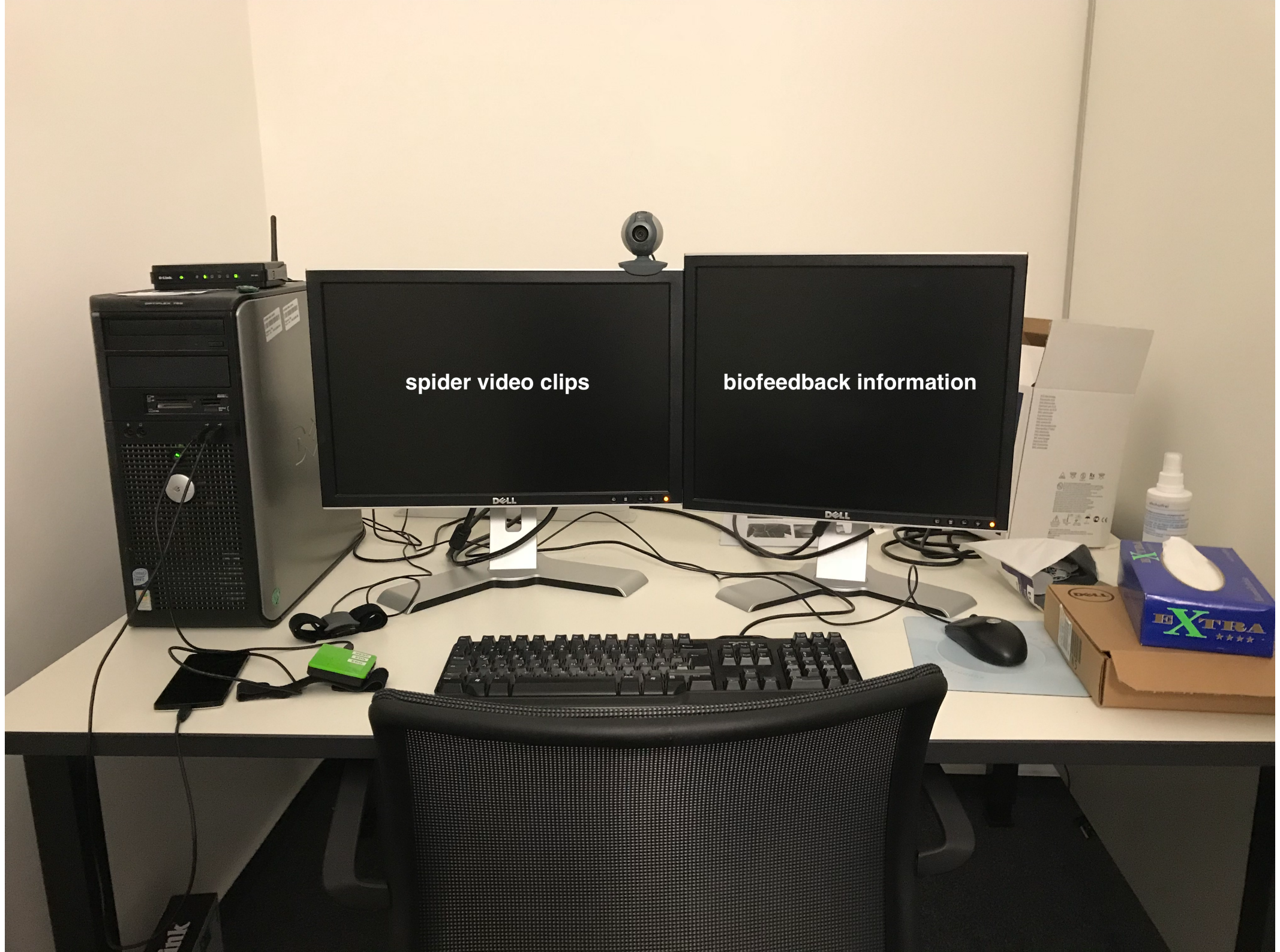

spider video clips

biofeedback information

Supplement: Supplementary file 2 — Photograph of the experimental set-up. (PDF 13386 kb) [file 13063_2018_2554_MOESM2_ESM.pdf]
