# Supplementary material for: Effects of heart rate variability biofeedback during exposure to fear-provoking stimuli within spider-fearful individuals: study protocol for a randomized controlled trial
Source: Trials. 2018 Mar 16;19:184. doi: 10.1186/s13063-018-2554-2 (PMC5857097; doi:10.1186/s13063-018-2554-2)

ADDITIONAL FILE 3: Effects of Heart Rate Variability Biofeedback during Exposure to Fear-Provoking Stimuli within Spider Fearful Individuals: Study Protocol for a Randomized-Controlled Trial

AUTHORS: Sarah K. Schäfer, Frank R. Ihmig, Karen A. Lara H., Frank Neurohr, Stephan Kiefer, Marlene Staginnus, Johanna Lass-Hennemann & Tanja Michael

I. HRV Biofeedback

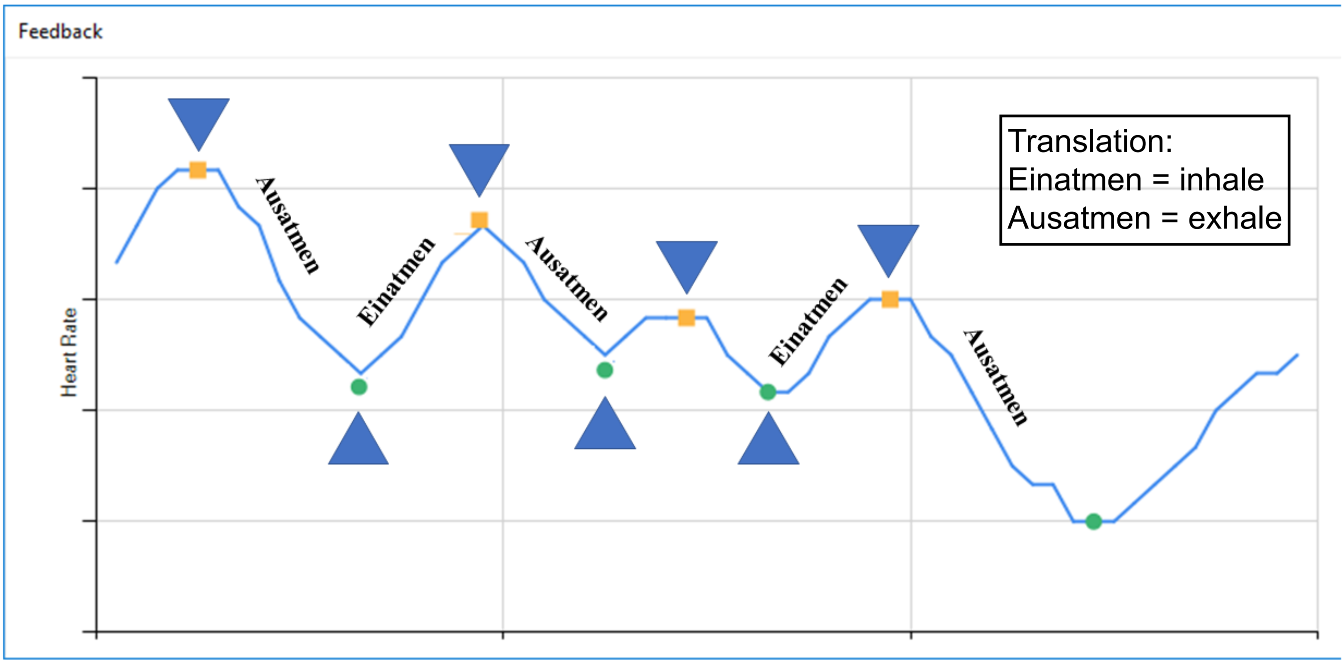

II. Tapping Task

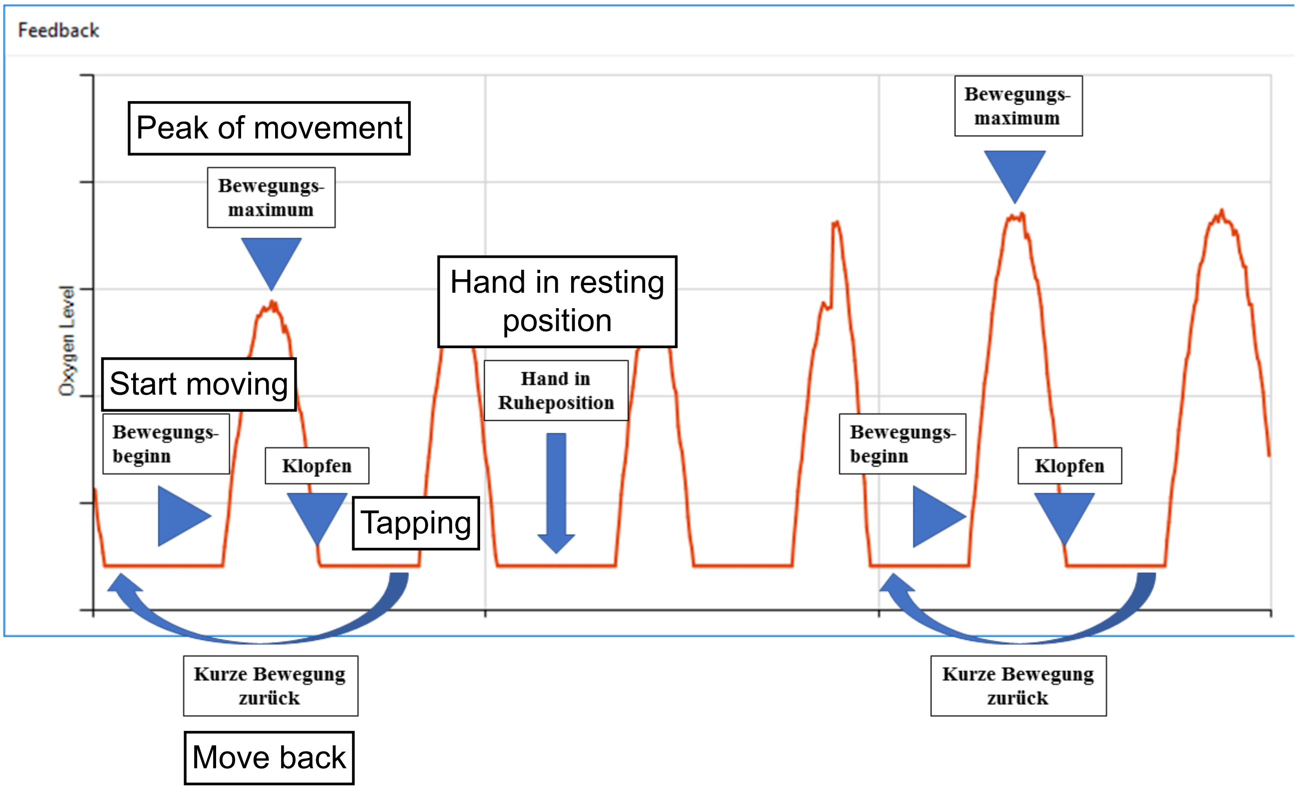

III. Rhythmic hand movement

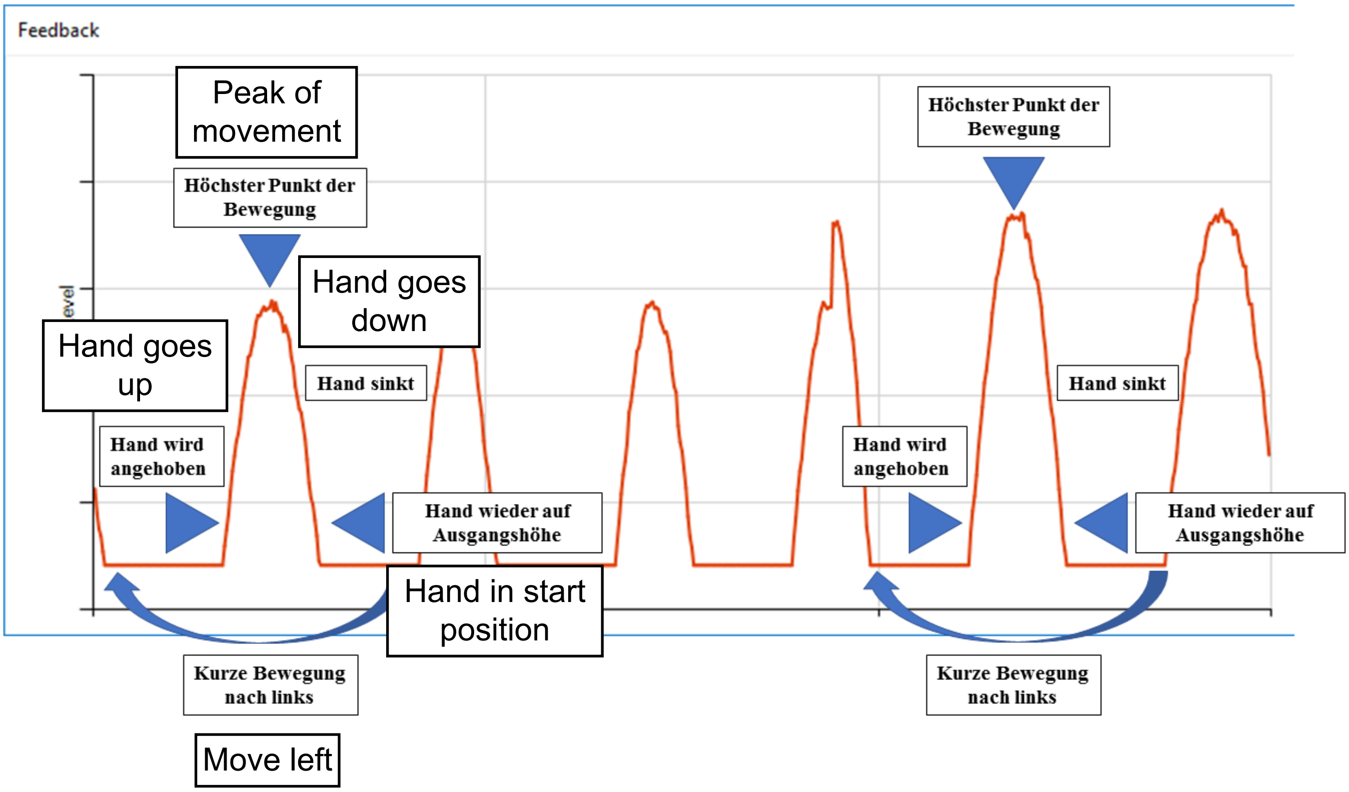

Supplement: Supplementary file 3 — Schematic diagram of heart rate variability (HRV) biofeedback and pseudo-biofeedback tasks. (PDF 387 kb) [file 13063_2018_2554_MOESM3_ESM.pdf]
